# Barriers to the Completion of Radiation Therapy in Cervical Cancer Treatment in Nigeria: A Review of Socioeconomic, Geographical, and Psychosocial Factors

**DOI:** 10.7759/cureus.70747

**Published:** 2024-10-03

**Authors:** Ajibola A Adebisi, Daniel E Onobun, Chijioke Orji, Reginald Ononye

**Affiliations:** 1 General Surgery, Royal College of Surgeons of England, London, GBR; 2 Orthopedics, South Warwickshire University NHS Foundation Trust, Coventry, GBR; 3 Orthopedics, Liverpool University Hospitals NHS Foundation Trust, Liverpool, GBR; 4 Urology, Glan Clwyd Hospital, Denbighshire, GBR

**Keywords:** cervical cancer, external beam radiotherapy, nigeria, pelvic radiation, radiotherapy

## Abstract

Cervical cancer is a major health concern in Nigeria, where it is one of the primary causes of cancer-related deaths among women. Despite the crucial role of radiation therapy in treating cervical cancer, many patients in Nigeria do not complete their prescribed treatment courses. This review examines the barriers contributing to this issue. Factors such as low awareness and utilization of Pap smears, inadequate HPV vaccination, financial constraints, insufficient radiotherapy infrastructure, and the psychological burden of cancer treatment are explored. Limited screening and vaccination efforts exacerbate the high incidence of cervical cancer in Nigeria. Financial barriers are a primary obstacle, with many patients unable to afford the high cost of radiotherapy. Nigeria's radiotherapy infrastructure is severely lacking leading to significant treatment delays and cancellations. Geographical barriers further complicate access, as many patients must travel long distances to reach treatment centers. Psychosocial issues, including anxiety and depression, significantly impact treatment adherence and completion. These psychological factors, coupled with the physical side effects of radiotherapy, contribute to high rates of treatment interruption. To address these challenges, the review suggests enhancing cervical cancer prevention through increased human papillomavirus (HPV) vaccination and screening, expanding radiotherapy capacity by increasing the number of treatment centers, and providing comprehensive support systems to address financial and psychosocial barriers. By implementing these strategies, it is possible to improve treatment adherence and outcomes for cervical cancer patients in Nigeria.

## Introduction and background

Cervical cancer remains a significant global health issue, particularly in developing countries. In 2020, the Global Burden of Cancer (GLOBOCAN) study reported that cervical cancer was the fourth most frequently diagnosed cancer and the fourth leading cause of cancer-related death among women, with an estimated 604,000 new cases and 342,000 deaths worldwide [1]. Cervical cancer is especially prevalent in 23 countries where it is the most diagnosed cancer and in 36 countries where it is the leading cause of cancer death. These regions include sub-Saharan Africa, Melanesia, South America, and South-Eastern Asia, with sub-Saharan Africa having the highest incidence and mortality rates [1]. According to the United Nations, cervical cancer is the third most common cancer in Nigeria and the second leading cause of cancer-related deaths among women aged 15 to 44. In 2020, Nigeria recorded 12,000 new cases and 8,000 deaths from cervical cancer [2]. In 2017, the Human Papilloma Virus (HPV) Information Center estimated that approximately 14,089 women in Nigeria are diagnosed with cervical cancer annually, with 8,240 dying from the disease, making it the second most frequent cancer among women aged 15 to 44 years [3].

Radiation therapy, including external beam radiotherapy and brachytherapy, is a standard treatment for cervical cancer patients, both as definitive and adjuvant therapy [4]. It utilizes ionizing radiation to destroy malignant cells. It is essential for Fédération Internationale de Gynécologie et d' Obstétrique (FIGO) stage 1b and above, which represents 60% of cervical cancer cases in developing countries as many as in developed nations [5]. Radiotherapy is indicated for 58% of cervical carcinoma cases [6]. Prolonged treatment is longer than 56 days per the American Brachytherapy Society guidelines [7]. This is of significant concern, especially in low and middle-income countries where cervical cancer alone accounts for 7% of patients needing radiotherapy [8]. Maranga et al. found that only 6.7% of cervical cancer patients received optimal combined external beam radiotherapy, brachytherapy, and adjuvant chemotherapy [9].

Despite the critical need for radiotherapy, many patients who start treatment do not complete the full course. Cohen et al. found that only 46.5% of cervical cancer patients at an urban tertiary health center in the United States completed treatment within the recommended timeframe of 56 days [10]. Various factors contribute to this issue, which will be discussed in this review.

## Review

Barriers to radiotherapy completion

High Burden of Cervical Cancer in Developing Countries

The highest regional incidence of cervical cancer cases is in sub-Saharan Africa per GLOBOCAN 2020 [1]. In sub-Saharan Africa, cervical cancer accounts for 22.5% of all cancer cases in women, with the majority of affected women residing in rural areas [11]. This finding aligns with the research by Jedy-Agba et al., which revealed that one-fourth of cancers in women from 2009 to 2010 in the Ibadan Cancer Registry were cervical in origin [12]. This high burden may be explained by the low awareness of Papanicolaou's smear, an important screening test for this condition.

Ayinde and Omigbodun reported that only 17% of female students at the University of Ibadan were aware of the screening test for cervical cancer (Papanicolaou’s smear), even though 81.5% of respondents were sexually active and only 8.3% had ever undergone a Papanicolaou's smear [13]. An earlier study by Ayinde and Omigbodun among female health workers in Ibadan found that 93.2% had never had a Pap smear, similar to findings by Idowu et al. in Ilorin, where only 8% had ever had a Pap smear [14]. Gabriel et al. found that only 18.2% of women accessing care in a rural tertiary hospital in Nigeria were aware of cervical cancer screening, and the Pap smear test utilization rate was 10.2% [15].

Almost all cervical cancer cases are linked to HPV infection [16], which is essential but not the only cause of cervical cancer [17]. The International Agency for Research on Cancer Monographs identifies 12 oncogenic HPV types as group 1 carcinogens, with HPV 16 and HPV 18 being the most significant [18]. These types are sexually transmitted and cause dysplastic changes in the cervix, which can lead to malignancy. Pap smears are effective in screening for these changes. In Nigeria, the HPV prevalence rate in women with normal cytology was 16% in 2012, and 66.9% of cervical cancer patients had HPV 16/18 [3]. Furthermore, HPV 16 and 18 were involved in 28.8% of high-grade pre-malignant cervical lesions. This highlights the importance of HPV vaccination and cervical cancer screening. Cervical cancer is uncommon in high-income countries largely due to the widespread use of Pap smears, which help in early detection and removal of precancerous lesions. However, in Nigeria, the low awareness and limited use of Pap smears hinder the detection of pre-malignant lesions, contributing to the higher incidence of cervical cancer [19]. The significant burden of cervical cancer in Nigeria and other developing countries indicates a greater need for radiotherapy. High-dose-rate (HDR) brachytherapy units, which can treat about 10-12 cases per day, are crucial [20]. However, due to the high patient volume, it is challenging for all cervical cancer patients requiring radiotherapy to receive full treatment within the recommended timeframe. A study by Anakwenze et al. found that 26% of chemotherapy patients at University College Hospital, Ibadan, faced delays because of high patient volume [21].

Inadequate Number of Radiotherapy Machines

The assertion that Africa "is functioning at 25% of its potential treatable capacity for cervical cancer alone" starkly illustrates the dire need for a more comprehensive global strategy in delivering radiation therapy [22]. Worldwide, about 56.4% of cancer patients have access to only 31.7% of the necessary teletherapy units. Within Africa, although 20 countries offer brachytherapy services, a staggering 75% of these services are concentrated in the northern regions and South Africa [23]. Ideally, the ratio of teletherapy units to individuals should be 1 per 120,000 to 250,000 people. In high-income countries, this ratio is around 1 unit per 130,000 people, whereas in low- and middle-income countries, it drastically drops to approximately 1 unit per 1.4 million people [24].

A 2014 study by Datta et al. assessed the radiotherapy infrastructure across 139 countries classified as low- and middle-income by World Bank standards. The findings were alarming: only 4 of these countries met their radiotherapy needs. On average, 80 countries had access to just 36.7% of the required radiotherapy services, and 55 countries had no access to radiation therapy whatsoever. Notably, 30 of these 55 countries were in Africa, with 60% of the continent's teletherapy units located in South Africa and Egypt [25]. Per Zubizarreta et al., as of 2015, low- and middle-income countries possessed around 4221 teletherapy units, a figure representing merely 38 to 49% of the total number needed [8].

In 2012, Nigeria alone accounted for 8.3% of the global cancer burden [26]. The International Atomic Energy Agency has identified Nigeria as having the largest gap between the availability of radiotherapy machines and the actual need for these services [21]. A 2015 radiotherapy needs assessment revealed that only 2 out of Nigeria's 9 radiation centers were operating at full capacity [26]. The country has approximately 1 radiation unit per 19.4 million people, a stark contrast to the recommended ratio of 1 unit per 120,000 to 250,000 people. Furthermore, research by Anakwenze et al. indicated that 91.3% of patients who completed radiotherapy treatments experienced delays or cancellations due to issues such as healthcare worker strikes, power failures, machine breakdowns, or prolonged wait times [21].

These findings underscore a critical issue: even when radiotherapy machines are available in low- and middle-income countries, the reliability and quality of access to this technology are inconsistent. This inconsistency is often due to challenges in maintaining and servicing the equipment properly. As a result, many patients in less developed nations do not complete their prescribed courses of radiation therapy, significantly impairing their treatment outcomes.

Low Socioeconomic Status

Parikh et al. have demonstrated that there is a significantly increased relative risk of developing cervical dysplasia and cervical cancer with decreasing social class [27]. Specifically, women belonging to the middle social class group exhibit an approximately 26% increased risk of cervical disease, while those in the lower social class tertile face an alarming 80% increased risk compared to their counterparts in the upper tertile [27]. Multiple studies have underscored low socioeconomic status as a critical determinant in cervical cancer screening rates. For instance, Idowu et al. conducted a study in Ilorin that revealed that women of lower socioeconomic status are less likely to undergo cervical cancer screening, thereby increasing their susceptibility to the disease [14]. Studies from high-income countries with advanced healthcare systems and more robust screening initiatives have also linked a lower socioeconomic status with an increased risk of developing either from decreased likelihood to attend a screening or other factors [28-30].

A 2017 report by the United Nations Development Programme (UNDP) highlighted that despite substantial economic progress over the past 25 years, illustrated by a robust Gross Domestic Product growth rate of around 5.0%, poverty levels in Africa remain exceedingly high at 41%, which is significantly higher compared to other developing regions [31]. The financial burden of curative radiotherapy for cervical cancer in sub-Saharan Africa is steep, with an average cost reported to be $1547.48 [32]. According to data from the World Resources Institute's environmental resource portal Earth Trends, approximately 71% of Nigerians live on less than $1 per day, and about 92% live on less than $2 per day. The poverty rate is estimated to have reached 38.9% in 2023, according to World Bank figures, making Nigeria home to the world’s second-largest poor population after India [33].

The high prevalence of cervical cancer among women of lower socioeconomic status is a matter of profound concern [27]. It is essential to recognize the pervasive impact of poverty on a significant portion of the African population, with poverty rates soaring as high as 41% [31], rendering many individuals unable to afford the prohibitive costs associated with curative radiotherapy. In a study by Anakwenze et al., it was revealed that a staggering 80% of patients at the University College Hospital in Ibadan were unable to bear the financial burden of radiotherapy without external assistance. Further exacerbating this issue, only 6% of these patients held federal insurance, which did not adequately cover the expenses of crucial radiotherapy services [21].

The situation in India is not far off. Findings by Dutta et al. showed that 87.5% of patients who completed their entire radiotherapy treatment within eight weeks were not struggling with impoverishment [34]. This revelation underscores the significant role that financial constraints play in the completion rates of treatment. It seems plausible that the inability to secure adequate funds may be a key factor contributing to the failure of many cervical cancer patients in Nigeria and across Africa to complete their radiotherapy treatment. Studies conducted in Africa have indicated that a lack of financial resources has led to decreased access to cervical cancer treatment, especially radiotherapy [35-39].

Given these findings, it is clear that economic barriers significantly hinder the ability of lower-income women to access and complete essential cervical cancer treatments. Urgent interventions are imperative, including the establishment of financial assistance programs, the subsidization of treatment costs, and the provision of comprehensive insurance coverage. These measures are essential to ensure that women across all socioeconomic strata can receive timely and comprehensive cancer care. Addressing these financial obstacles is crucial for improving health outcomes and for stemming the incidence and mortality rates associated with cervical cancer in low-income populations.

Distance From the Treatment Center

The accessibility of radiotherapy is a significant challenge for many patients who often have to travel long distances to reach treatment centers [21]. This problem is not limited to Nigeria or sub-Saharan Africa. For example, a study by Dutta et al. found that nearly two-thirds of patients in India lived far from major towns and had to travel five to six hours by train or road to access treatment facilities [34]. Similar transportation and distance-related issues have been reported in studies conducted in Houston, Texas, and Chicago [40,41].

Interestingly, Cohen et al. discovered that patients living closest to treatment centers were the least likely to complete radiotherapy within the recommended timeframe. This was attributed to the treatment centers being situated in urban, low-socioeconomic areas, which may present additional barriers to consistent treatment adherence [10]. That being said, more often than not, the completion rate of the full treatment course for cervical cancer requiring chemoradiation tends to be lower for patients living farther from the treatment centers [42].

The geographical and logistical barriers to accessing radiotherapy are further compounded by the socioeconomic conditions of the patients. Many individuals in low- and middle-income countries face additional hardships such as poor road infrastructure, limited availability of public transportation, and financial constraints, making long-distance travel for medical treatment particularly burdensome. This situation not only delays the initiation of treatment but also increases the likelihood of interruptions and non-completion of the prescribed therapy regimen.

Psychosocial Problems

Cancer is a devastating disease that brings significant psychological challenges not only to the patients but also to their families [43]. Patients often rely on caregivers who provide essential psychosocial support and care. Despite this support, cancer patients continue to face numerous psychological hurdles. A study conducted at a comprehensive cancer center in the United States involving nearly 4,500 patients aged 19 and older found that the prevalence of significant psychological distress ranged from 29% to 43% among patients with the 14 most common types of cancer [43]. These findings align with subsequent studies across diverse cancer populations, which report high rates of psychological symptoms that meet the criteria for clinical diagnoses such as depression, adjustment disorders, and anxiety [44-46].

Radiotherapy can exacerbate these psychological issues. It is associated with long-term physical side effects, such as pain and reduced physical functioning, as well as emotional distress, including anxiety and depression [47]. These psychological factors can significantly influence the treatment trajectory for cancer patients, affecting both their quality of life and their ability to adhere to treatment protocols. Various studies have highlighted that anxiety and depression are prevalent and significant issues that impact the overall well-being of cancer patients. These conditions diminish the quality of life, reduce treatment compliance, and extend hospitalization periods [48,49].

The psychological burden of cancer is profound. Beyond the immediate impact of the disease, the treatment process itself can be mentally and emotionally taxing. The anticipation of radiotherapy, the experience, and the side effects all contribute to heightened stress levels. Patients often grapple with fears about treatment efficacy, potential side effects, and the overall prognosis. This psychological stress can manifest in various ways, including sleep disturbances, appetite changes, and mood swings.

Research by Cohen et al. identified underlying psychological factors as major barriers to the timely completion of radiation therapy in cervical cancer patients [10]. Additionally, Zaki et al. found that in 59% of cervical cancer patients who experienced prolonged treatment durations, the primary causes were social issues and non-adherence to treatment protocols [7]. Despite these findings, there is a notable lack of local studies in the Nigerian context that specifically examine the correlation between psychosocial issues and the extended duration of radiation therapy in cervical cancer patients.

Radiation Toxicity

Radiation toxicity is a well-documented side effect of radiation therapy. While radiation therapy is effective because it damages the DNA of cancerous cells, the irradiation of surrounding normal tissues can also lead to toxicity. Radiation toxicity is generally classified into acute toxicity, which occurs during treatment or within weeks of treatment, and late toxicity, which manifests after six months to several years post-treatment. Late effects can be particularly concerning as they may be irreversible and can limit the doses used in radical radiotherapy regimens. Hence, it is essential to monitor late toxicity rigorously to fully assess the therapeutic benefits of treatment, necessitating prolonged follow-up periods [50].

Acute radiation toxicity can present with a range of symptoms, including nausea, vomiting, mouth sores, proctitis, cystitis, and infertility. These symptoms can significantly affect a patient's quality of life during and immediately after treatment. On the other hand, late radiation toxicity includes conditions such as lymphedema, hair loss (epilation), chronic radiation enteropathy, contractures, gynecologic fistula, and vaginal stenosis. These late effects not only impact long-term health but also necessitate continuous medical management.

In a study conducted by Roszak et al., it was found that the incidence of acute toxicity in patients undergoing pelvic irradiation was as high as 51.3%, while the incidence of late toxicity reactions was 14.8% [4]. The study highlighted that gastrointestinal tract toxicity was more prevalent than urinary system toxicity. Notably, early bladder toxicity was most common among cervical cancer patients who had undergone surgery [4]. The research also indicated that delays in treatment were associated with higher rates of toxicity.

Radiation toxicity's impact on treatment completion is a critical concern. Acute symptoms can be debilitating, leading some patients to interrupt or cease treatment prematurely, which can adversely affect the overall success of therapy. The long-term complications of late toxicity further complicate patient management, as they require ongoing care and can significantly diminish a patient's quality of life.

Although these findings provide valuable insights into the general effects of radiation toxicity, there is a notable gap in the literature regarding the specific relationship between radiation toxicity and the completion of radiotherapy treatment in Nigeria. This gap underscores the need for localized studies to understand better how these side effects influence treatment adherence and outcomes in different settings.

Way forward

While this review has identified several factors contributing to the prolongation and failure to complete radiation therapy among cervical cancer patients in Nigeria, it is crucial to recognize that many of these challenges can be addressed. This section will propose potential solutions to mitigate these issues.

Cervical Cancer Prevention: Screening and Vaccination

The high incidence of cervical cancer in Nigeria is largely due to low levels of awareness, inadequate screening, and insufficient vaccination against HPV. Since HPV is responsible for the majority of cervical cancer cases and is a sexually transmitted infection, it is recommended that females be vaccinated against HPV before their sexual debut. Currently available vaccines include Gardasil (quadrivalent), Gardasil 9 (9-valent), and Cervarix (bivalent). Through widespread vaccination, cervical cancer rates could be reduced by 70% to 90% over the next 25 years [51].

Canada's experience demonstrates the effectiveness of vaccination programs. Cervical cancer mortality in Canada decreased from 13.5 to 2.2 per 100,000 (an 83% reduction) between 1952 and 2006, primarily due to vaccination efforts [52]. Most Canadian provinces offer free HPV vaccinations to females aged nine to 17 years, a strategy that could be adopted in Nigeria and other sub-Saharan African countries. Evidence from a study by Bird et al. indicates that vaccination uptake is five times higher when provided free of charge compared to out-of-pocket payment [53]. Integrating HPV vaccination into school health programs and launching comprehensive awareness campaigns about cervical cancer could significantly reduce the need for radiotherapy and associated wait times.

Increasing the Number of Radiotherapy Centers

Nigeria faces a significant treatment gap, with only one radiation machine available for every 19.4 million patients, far below the recommended ratio of 1:120,000-250,000 [19]. Increasing the number of radiotherapy machines and centers is essential to bridge this gap, reduce wait times, and ensure timely treatment for cervical cancer patients. Expanding the distribution of treatment centers across the country would also minimize the distance patients must travel to access care, thereby reducing the incidence of treatment delays and prolongation.

## Conclusions

This review has highlighted several factors that contribute to treatment delays and incomplete radiation therapy among cervical cancer patients in Nigeria. Financial constraints are the primary challenge for most patients, making it difficult for them to access and complete treatment. Additionally, the insufficient number of radiotherapy machines, leading to long wait times, poses a significant barrier. Other issues, such as radiation toxicity, psychosocial problems, and geographical challenges related to the distance from treatment centers, further complicate treatment completion.

Addressing these challenges requires a multifaceted approach. Key strategies include enhancing prevention efforts through widespread HPV vaccination and comprehensive screening programs to reduce cervical cancer incidence. Expanding radiotherapy capacity by increasing the number of machines and treatment centers is crucial to ensure timely access for all patients. Furthermore, improving support systems by offering financial assistance, psychological support, and patient education can help enhance adherence to treatment protocols. Local studies are necessary to further explore the specific impacts of these factors on treatment completion in Nigeria, allowing healthcare providers to develop targeted interventions that improve outcomes for cervical cancer patients and increase radiotherapy completion rates.
